# Human T Lymphotropic virus-1 associated gastrointestinal histoplasmosis in Peru

**DOI:** 10.3855/jidc.1030

**Published:** 2011-07-04

**Authors:** Carlos Canelo-Aybar, Jose Cuadra-Urteaga, Fernando Atencia, Franco Romani

**Affiliations:** 1 Department of Internal Medicine, Arzobispo Loayza Hospital, Lima, Peru; 2 Faculty of Medicine, San Marcos University, Lima, Peru

**Keywords:** gastrointestinal histoplasmosis, HTLV-1, immunology, Peru

## Abstract

We report a 72-year-old patient with chronic diarrhoea and histologic evidence of gastrointestinal histoplasmosis. He had no history of HIV or of taking immunosuppressive drugs. The patient was found to be a carrier of Human T-lymphotropic virus-1, a condition associated with inflammatory, lymphoproliferative, and opportunistic infectious diseases. To our knowledge, there are only three previous cases reporting this coinfection and this is the first documented case with gastrointestinal involvement.

## Introduction

Histoplasmosis is a systemic fungal disease caused by *Histoplasma capsulatum*, a dimorphic and ubiquitous fungus. The disease is endemic in certain areas of North, Central, and South America, as well as Africa and Asia [1]. In Peru, *H. capsulatum* is most prevalent in tropical forest areas, where up to 43% of seroprevalence to *H. capsulatum* has been reported [2, 3]. Most primary infections with *H. capsulatum* are either asymptomatic or result in mild influenza-like illness; however, certain forms of histoplasmosis can cause life-threatening infections with considerable morbidity [1, 4, 5]. Gastrointestinal histoplasmosis (GH) usually develops in the context of a disseminated infection and it is associated with severely immunosuppressed patients, especially those with AIDS [5, 6].

Human T-lymphotropic virus 1 (HTLV-1) is a retrovirus of global distribution, endemic in Japan, sub-Saharan Africa, the Caribbean and South America, where more than 1% of the general population is infected [7]. In Peru, prevalence has varied from 1.3% to 3.8% in studies from coastal and highland regions [8]. Chronic infection with HTLV-1 is associated with diverse diseases that can be divided into three categories: 1) neoplastic diseases (adult T-cell leukemia /lymphoma); 2) inflammatory syndromes (HTLV-1 associated myelopathy/tropical spastic paraparesis); and 3) opportunistic infections secondary to an impaired immune response, due to organisms such as *Strongyloides stercoralis, Mycobacterium tuberculosis*, and *Sarcoptes scabiei* [7]. Although it has been estimated that the life-time risk of developing any of the HTLV-1 associated diseases could approximate 10%, the vast majority of people remain asymptomatic [7].

We report a case of GH associated with HTLV-1 infection. To the best of our knowledge, it is the fourth case of HTLV-1 and *H. capsulatum* coinfection reported in the literature and the first one with gastrointestinal involvement.

## Case report

A 72-year-old male patient with no previous medical history was admitted at Arzobispo Loayza Hospital in November 2007, after moving to Lima from Tarapoto, a tropical forest region of Peru. For approximately one year he had intermittent episodes of diarrhoea without blood or mucus, which progressively worsened to persistent diarrhoea accompanied by hyporexia, nausea, and oral intolerance. During the two months prior to the onset of gastrointestinal symptoms, he was febrile (38-39 °C) every other day and had marked weight loss.

Physical exam on admission revealed a cachectic man with normal vital signs with the exception of fever (39°C). He was found to be alert, anicteric, chronically ill and emaciated. The liver was found to be two centimeters below the right ribe border, and no palpable masses were detected. Edema with fovea in the lower limbs was noted. His laboratory tests revealed the following abnormal results: hemoglobin 8.38 gr/dL, albumin1.93 gr/dL, globulins 2.99 gr/dL, prothrombin time 60 seconds, and platelet count 75,000 µ/L. Giemsa-stained blood smears for *Plasmodium sp*. and *Bartonella bacilliformis*, and Ziehl-Neelsen stains of sputum and feces for evidence of mycobacteria were negative. Serologic tests were negative for *Echinococcus granulosus, Leptospira, Paracoccidioides brasiliensis, Toxoplasma gondii*, and, notably, *H. capsulatum*.

The case patient tested negative for HIV using the The ARCHITECT HIV Ag/Ab Combo assay (Abbott Diagnostics, IL, USA), which is a chemiluminescent microparticle immunoassay for the simultaneous detection of HIV p24 antigen and antibodies. However, the patient’s result was positive for antibodies against HTLV1/2, using the ARCHITECT rHTLV-I/II (Abbott Diagnostics) assay for detection of antibodies against recombinant antigens (gp21 from HTLV-2) and synthetic peptides (gp46 from HTLV-1/2).

**Figure 1:**
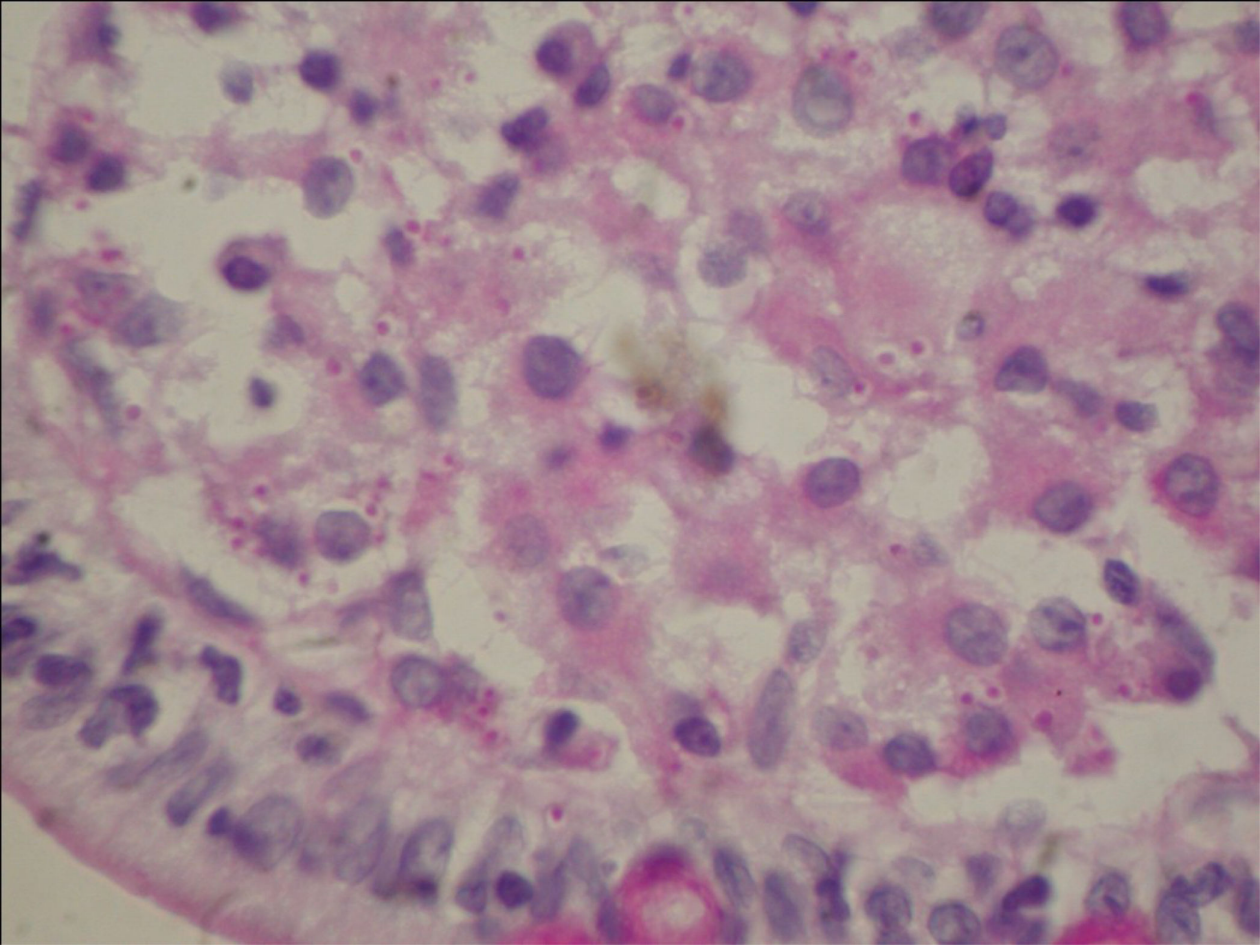
Disseminated histoplasmosis of the small bowel. The section revealed macrophages in the lamina propia which contains small budding yeast 2-4 µm in diameter (PAS 40X magnification).

**Figure 2:**
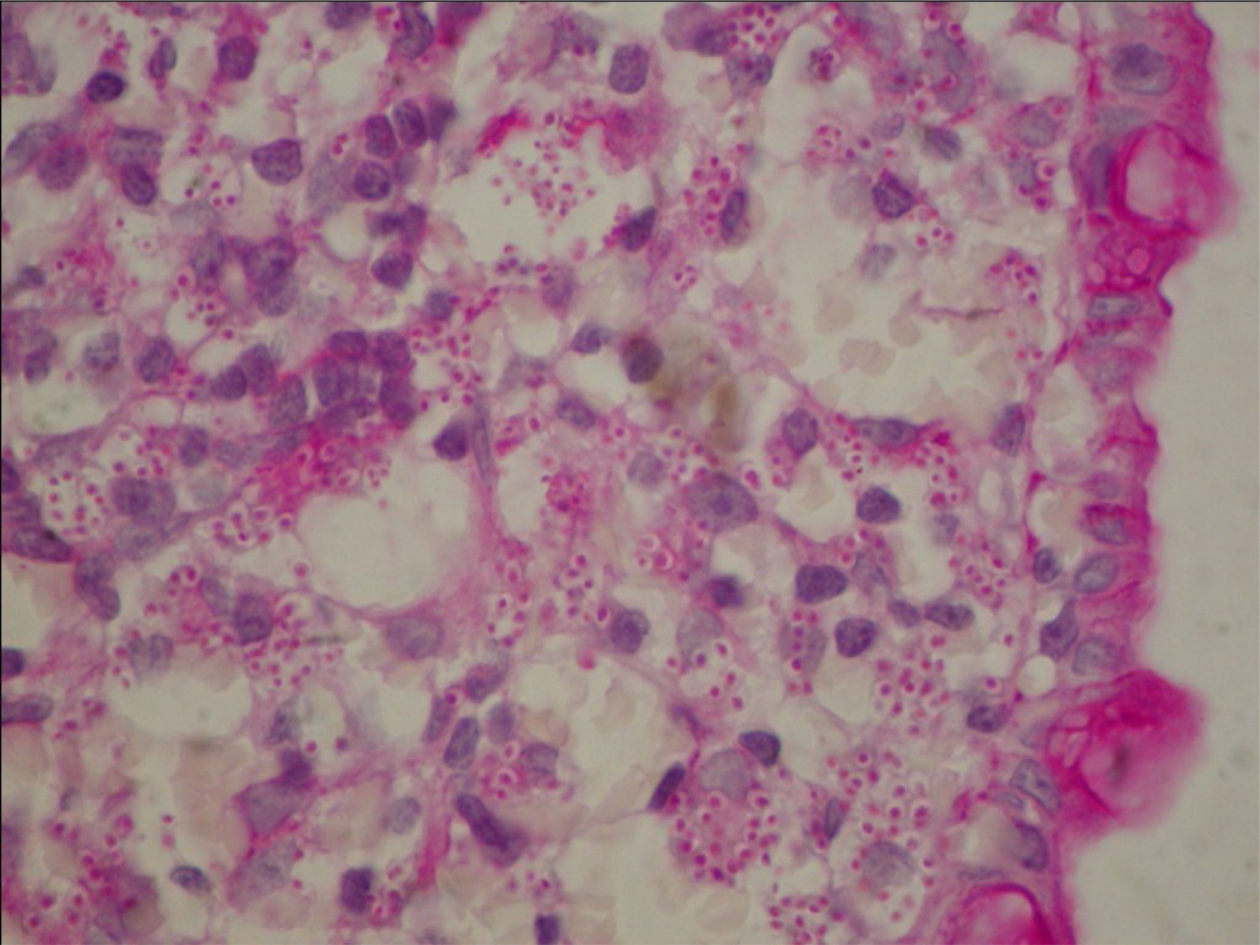
Disseminated histoplasmosis of the colon. The specimen revealed myriads of budding yeast 2-4 µm in diameter within the lamina propia, the basophilic cytoplasm is retracted from the wall of the yeast, producing a “halo like” appearance (PAS 40X magnification).

An abdominal ultrasound showed a 40 mm mass in his right flank. Contrast radiographic intestinal assessment showed fragmented and disorganized progression of the barium suspension to the jejunum and partially to the ileum. The upper endoscopy procedure showed superficial gastritis with deformation of the duodenum folds and the colonoscopy showed multiple ulcerative lesions of the proximal colon with ileal involvement. Multiple biopsies were taken during both exams and results revealed partial disruption of the third portion of the duodenum. Macrophages were found to contain intracytoplasmic round microorganisms 2-4 µm in diameter that had capsules and cell nuclei suggestive of *H. capsulatum* (Figure 1). A biopsy of the colon also revealed partially disrupted and ulcerated mucosa with macrophages containing the same intracellular microorganisms found in the duodenum (Figure 2). The specimens were not sent to either the Microbiology or Mycology Department for culture because *H. capsulatum* was not in the differential diagnosis at that time.

The biopsies were consistent with the diagnosis of GH and consequently the patient was treated with amphotericin B at a dosage of 1 mg/kg/day. Unfortunately, after five days of treatment, the patient developed respiratory insufficiency and leukocytosis. The presence of bilateral alveolar infiltrates that were not observed upon intial presentation were noted on chest X ray. The results of blood cultures that were taken to rule out a bloodstream infection were negative. The patient died from irreversible shock nine days after the diagnosis was made. His relatives refused to authorize an autopsy.

## Discussion

*H. capsulatum* was first described as a cause of disease by Darling in 1906 [9]. The natural habitat of this fungus is soil that has been contaminated with bird or bat droppings. Pulmonary infection usually develops through inhalation [1, 3], followed by haematogenous spread to the reticuloendothelial system within a few weeks before the onset of specific cellular immunity [1, 5].

The majority of individuals newly infected with *H. capsulatum* remain asymptomatic and fewer than 5% complain of mild subacute pulmonary symptoms weeks to months following exposure [1, 5]. However, patients with underlying conditions that impair the immune system develop progressive disseminated histoplasmosis (PDH), which is defined as a clinical illness associated with extrapulmonary tissue involvement that does not improve after at least three weeks of observations [4]. Reported risk factors for PDH include: AIDS, primary immunodeficiency, immunosuppressive medications such as corticoids, methotrexate, TNF-alpha inhibitors, and advanced age [1, 4–6].

Approximately 70% of patients with PDH show gastrointestinal disease at autopsy; however, this is rarely recognized because only 3-12% of patients are symptomatic [2, 4–6]. Among symptomatic individuals, 30-50% are febrile and complain of weight loss or diarrhoea [1, 4–6]. Upon physical examination, splenomegaly or hepatomegaly is found in 30 – 100% of cases [5] and abdominal lymphadenopathy is described in 66% of CT scans [5]. Gastric involvement is found in only 4% of autopsies and intestinal complications may vary from gastrointestinal bleeding, perforation, and peritonitis to malabsorption syndrome [4]. Inflammatory masses mimicking a malignancy have been described in immunosuppressed patients with colonic obstruction [10]. Small bowel obstruction with segmental strictures and ulcerations has also been reported [11].

GH in our patient was characterized by chronic diarrhoea, weight loss, fever, and ulcerations involving the ileocecal segment. Although the entire gastrointestinal tract may have been involved, the distal ileum segment was the region predominantly affected, as indicated by the abundance of lymphoid tissue in this area [6].

A variety of diagnostic exams for histoplasmosis have been described with diverse results. In general, serology tests for the detection of antibodies are less sensitive in patients with disseminated disease or severe immunosuppression [5], which may explain the sero-negativity of our case patient. Histoplasma antigens can be detected in the urine of 90% of patients with PDH, but false-negative results are common when the disease is localized in areas such as gastrointestinal mucosa [1, 4–6]. Although cultures are positive in about 85% of PDH, multiple tissue or blood culture specimens are required to achieve high sensitivity and 4-6 weeks are required for definitive results [5].

Histopathology provides a rapid diagnosis but is less sensitive than culture and requires the diagnostic skills of an experienced pathologist [1, 4]. Pathologically four forms of intestinal histoplasmosis have been described as follows: 1) no gross abnormalities at endoscopy, fungal forms observed within the lamina propia; 2) small pseudopolyps and plaques caused by conglomerates of infected macrophages; 3) ulceration with tissue necrosis; and[4) localized inflammation [6]. In tissue sections, *H. capsulatum* is often found in clusters within the cytoplasm of macrophages and appears as uniform ovoid-to-spherical 2-4 µm uninucleate yeast with narrow base buds [5].

The HTLV-1 virus, first isolated in 1979 from a patient with a T-cell malignancy [7], is endemic in Peru with a reported prevalence of 2.3% in pregnant women from Quillabamba and 18.6% in HIV-positive men from Lima [8]. On the other hand, the rare reports of HTLV-2 infections in Peru have included the following: 2 of 64 (3.1%) of indigenous Peruvians living in the Amazon Jungle [12]; 35 of 2,703 (1.3%) and men who have sex with men. Of interest, no HTLV-2 infections were detected among 200 female sex workers in Iquitos [13].

Confirmatory testing with line immunoassays, Western Blot immunofluorescence assays or molecular methods are recommended; however, these tests are expensive and often not accessible in developing countries. A recent study in Lima, Peru, was undertaken to determine the positive predictive value of three different ELISA assays and their accuracy in differentiating between HTLV-1 and HTLV-2 infections [14]. No false-positive results were found and results showed that 98.93% of cases were HTLV-1 infected [14]. These findings indicate that an accurate diagnosis of HTLV-1 can be made with an ELISA test in a high-prevalence setting.

The pathogenic effect of HTLV-1 on the immune system is associated with a predominant Th1 response with a high amount of IFN-γ production, which predisposes infected individuals to helmintic infections such as *S. stercoralis* 15]. This effect, however, does not explain the predisposition of patients to developing fungal infections. HTLV-1 can have a detrimental effect on cellular immunity by affecting the interaction between T cells and macrophages, as suggested by the high prevalence of tuberculosis among people infected with HTLV-1 and a reduced delayed-type hypersensitivity response to PPD [16]. The mechanism of such immunosuppression remains unknown, but may provide a plausible explanation for the association between HTLV-1 and *H. capsulatum* coinfection.

There are only three previous cases with HTLV-1 and *H. capsulatum* coinfection reported in the literature: the first was a lymphoma with multiple opportunistic infections (*H. capsulatum* in addition to *Pneumocystis jirovecii, S. stercoralis, Giardia lamblia*, and *Cytomegalovirus*) [17]; the second was pulmonary histoplasmosis with a right upper lobe cavitary lesion [18]; and the third was disseminated histoplasmosis with spinal chordoma, brain granulomas, and submaxillary adenitis. In all cases there was no evidence of gastrointestinal dissemination [19]. Our case and the previous reports suggest a relationship between HTLV-1 infection and histoplasmosis. Interestingly, the severity and presentation of the gastrointestinal complications in the patient described here has only been reported in patients with severe immunosuppression. This may suggest that the effect of HTLV-1 on the immune system in this case report was more profound than usual.

In severe untreated disseminated histoplasmosis mortality is about 80%, but treatment with amphotericin B reduces mortality to 25% [4]. Although the patient presented here died from irreversible shock, the etiologic cause of death was not determined. Considering the negative results of the sputum and blood cultures, and the temporal association with amphotericin exposure, an inflammatory response to therapy cannot be disregarded. In addition, the possibility of pulmonary infection should also be considered.

In summary, we conclude that the diagnosis of GH is probably underreported in Peru, as exemplified by the high prevalence of *H. capsulatum* infection in certain areas in Peru and in other countries. The clinical manifestations of HTLV-1 infection are frequently unrecognized by general practitioners and the pathogenic mechanisms remain poorly understood due to the diverse effects of this virus on the immune system. From a public health perspective, there must be an increased awareness in developing countries of the association of HTVL-1 carriers with increased susceptibility to contracting histoplasmosis and other infectious diseases in both apparently healthy or immunosuppressed individuals.
